# Actin-dependent activation of serum response factor in T cells by the viral oncoprotein tip

**DOI:** 10.1186/1478-811X-10-5

**Published:** 2012-03-03

**Authors:** Kristin Katsch, Sarah Jill de Jong, Jens-Christian Albrecht, Julia Steger, Harald Genth, Guido Posern, Brigitte Biesinger

**Affiliations:** 1 Institut für Klinische und Molekulare Virologie, Friedrich-Alexander-Universität Erlangen-Nürnberg, Erlangen, Germany; 2 Institut für Toxikologie, Medizinische Hochschule Hannover, Hannover, Germany; 3 Department Molecular Biology, Max-Planck-Institut für Biochemie, Munich, Germany

**Keywords:** Actin, Herpesvirus saimiri, Lck, MRTF, Oncoprotein, Serum response factor, T lymphocyte

## Abstract

Serum response factor (SRF) acts as a multifunctional transcription factor regulated by mutually exclusive interactions with ternary complex factors (TCFs) or myocardin-related transcription factors (MRTFs). Binding of Rho- and actin-regulated MRTF:SRF complexes to target gene promoters requires an SRF-binding site only, whereas MAPK-regulated TCF:SRF complexes in addition rely on flanking sequences present in the serum response element (SRE). Here, we report on the activation of an SRE luciferase reporter by Tip, the viral oncoprotein essentially contributing to human T-cell transformation by Herpesvirus saimiri. SRE activation in Tip-expressing Jurkat T cells could not be attributed to triggering of the MAPK pathway. Therefore, we further analyzed the contribution of MRTF complexes. Indeed, Tip also activated a reporter construct responsive to MRTF:SRF. Activation of this reporter was abrogated by overexpression of a dominant negative mutant of the MRTF-family member MAL. Moreover, enrichment of monomeric actin suppressed the Tip-induced reporter activity. Further upstream, the Rho-family GTPase Rac, was found to be required for MRTF:SRF reporter activation by Tip. Initiation of this pathway was strictly dependent on Tip's ability to interact with Lck and on the activity of this Src-family kinase. Independent of Tip, T-cell stimulation orchestrates Src-family kinase, MAPK and actin pathways to induce SRF. These findings establish actin-regulated transcription in human T cells and suggest its role in viral oncogenesis.

## Background

Serum response factor (SRF) is widely expressed in both invertebrates and vertebrates. SRF plays an essential role in embryogenesis, but is also involved in multiple processes in developed organisms including neuronal and muscle cell function.

SRF binds as a dimer to a specific DNA sequence known as the CArG box in the promoter of hundreds of target genes. Selective binding is determined by interactions with more than 60 different cofactors, which turn SRF into a versatile transcription factor translating cell- and stimulus-specific signaling into selective target gene expression [1,2].

Well-known SRF cofactors are members of the ternary complex factor (TCF) family of Ets domain proteins, like Elk-1, SAP-1 and Net. They are regulated by phosphorylation via the classical mitogen-activated protein kinase (MAPK) pathway involving the GTPase Ras, which activates the serine-threonine kinases Raf, MEK and ERK. Their recruitment to DNA depends on a defined DNA sequence, called Ets motif (C/A)(C/A)GGA(A/T), next to the SRF-binding CArG box [3,4]. A serum response element (SRE), first described in the *c-fos *promoter, contains an Ets motif adjacent to the CArG box [5].

Another group of SRF cofactors are the myocardin-related transcription factors (MRTFs). Myocardin, the founding member of this family, is selectively expressed in cardiac and smooth muscle cells and constitutively binds SRF. In contrast, MRTF-A (MAL, MKL1, BSAC) and MRTF-B (MAL16, MKL2) are widely expressed in many cell types [6]. Their cofactor function is controlled by GTPases of the Rho family (RhoGTPases), which are considered as important regulators of the actin cytoskeleton. Activation of the RhoGTPases RhoA, Rac1 and Cdc42 results in the formation of focal adhesion complexes, lamellipodia and filopodia, respectively [7]. These processes involve actin polymerization and thereby reduce the levels of monomeric, globular actin (G-actin). G-actin binds to N-terminal RPEL motifs of MRTF and thereby sequesters and negatively regulates MRTF. RhoGTPase-mediated reduction of G-actin liberates MRTF, resulting in its nuclear accumulation and SRF cofactor function. SRF-bound MRTF dimers directly contact DNA near the SRF binding sequence. However, a specific MRTF binding sequence, similar to the Ets motif, has not yet been found [1,6].

Differential regulation of SRF target genes is based on gene-specific cofactor preferences and cofactor competition for a common binding site on SRF [8-11]. In this context, specific SRF functions are defined only for a limited set of cell types and assignment of cofactors is lagging. Conditional knock-out approaches were recently used to elucidate the function of SRF and the role of TCFs and MRTFs in mouse T cells. Elimination of SRF by a CD4-Cre transgene at the CD4^+^CD8^+ ^double positive stage impairs T-cell development and results in the absence of peripheral T cells [12]. An earlier elimination of SRF by a hCD2-Cre transgene at the CD4^-^CD8^- ^double negative stage severely reduces the numbers of single positive thymocytes, thymic T_reg _and NK T cells. Introduction of recombinant SRF lacking the ability to bind TCFs or MRTFs fails to restore thymocyte maturation. In contrast, reconstitution was successful upon introduction of wild-type SRF or a fusion of the recombinant SRF with Elk [13]. While this study documents an essential role of TCF:SRF complexes in T-cell development, activation and function of MRTF:SRF complexes in T cells remain to be established.

Herpesvirus saimiri (HVS) is the T-lymphotropic prototype of γ2-herpesviruses. In contrast to the apathogenic appearance in its natural host, the squirrel monkey (*Saimiri sciureus*), HVS causes severe T-cell lymphoma in experimentally infected non-natural primate hosts [14]. Most notably, *in vitro *infection of human peripheral blood mononuclear cells with HVS strain C488 gives rise to continuously proliferating T-cell lines [15]. Deletions of viral genomic sequences coding for the oncoproteins StpC (Saimiri transformation-associated protein of subgroup C) and Tip (Tyrosine kinase interacting protein) obviate human T-cell transformation as well as pathogenicity in non-human primates [16]. Conditional expression of Tip alone in transgenic mice leads to T-cell lymphoma [17]. Tip engages the Src-family kinase (SFK) Lck, a central mediator of proliferation in response to T-cell receptor stimulation [18,19]. Lck interaction and activation relies on two motifs in Tip, a sequence homologous to the C-terminus of Src-family kinase domains (CSKH) and a proline-rich Src homology domain 3 binding sequence (SH3B) [18,20,21]. The integrity of both motifs, CSKH and SH3B, is required for Tip to support human T-cell transformation [22]. However, pro-proliferative downstream effectors of Tip:Lck interaction are not defined yet. Pro-oncogenic functions are characterized for signal transducer and activator of transcription 3 (STAT3) [23]. Indeed, STAT3 is activated by Lck in the presence of Tip and is constitutively phosphorylated in HVS-C488 transformed lymphocytes [21,24-26]. However, mutation of tyrosine residue 114 (Y114) in Tip abrogates constitutive STAT3 phosphorylation, but not viral transformation of human T cells [27,28]. Thus, alternative Tip:Lck effectors must be involved to trigger T-cell proliferation. Given the central role of mitogen-activated protein kinases (MAPK) for growth regulation in general, we previously analyzed MAPK phosphorylation and activation of MAPK-regulated transcription in the presence of the HVS-C488 oncoproteins, StpC and Tip [29]. In Jurkat T cells, neither StpC nor Tip induce the phosphorylation of MEK1/2 and ERK1/2 or the activity of the MAPK-regulated transcription factor AP-1. Nevertheless, Tip specifically triggers SRF activity in this test system [29].

In this work, we now address the mechanism of SRF activation by the viral oncoprotein Tip. We demonstrate an SRF activation in T cells that depends on actin polymerization and on the cofactor MAL and is abrogated by dominant-negative Rac1. Tip requires Lck interaction and Src kinase activity to induce this pathway, which may also be a target of T-cell receptor stimulation.

## Results

### Tip induces SRF-regulated transcription independent of MAPK activity

**Figure 1:**
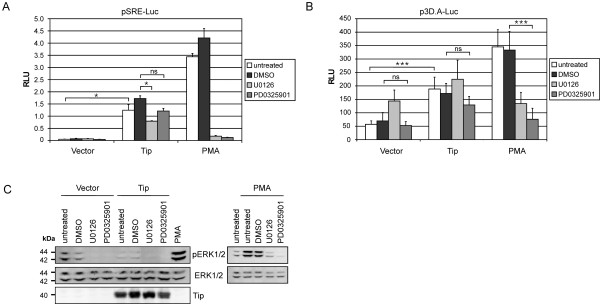
**Tip activates SRF independent of MAPK**. Jurkat T cells were transiently transfected with expression plasmids coding for the viral oncoprotein Tip of HVS-C488. Empty vector served as a negative control. For inhibition of MAPK signaling, cells were left untreated or treated with U0126 (25 μM), PD0325901 (1 μM) or solvent (DMSO) for 40 h. As positive controls, vector-transfected cell were stimulated with PMA (20 ng/ml) in the absence or presence of the MEK inhibitors for 15 h. **(A) **Activity of cotransfected pSRE-Luc displayed as mean values and standard deviations of an assay performed in triplicates, which is representative for three independent experiments. Statistical significance for correlated samples, p < 0.05 (*); p > 0.05 (ns). **(B) **Activity of cotransfected p3D.A-Luc summarized from six independent experiments. Statistical significance for independent samples, p < 0.001 (***); p > 0.05 (ns). **(C) **Verification of MEK inhibition by detection of ERK1/2 phosphorylation and expression as well as control of Tip expression by immunoblot analyses with whole cell lysates obtained 48 h post transfection

We previously reported activation of a serum response element (SRE) luciferase reporter by the viral oncoprotein Tip in Jurkat T cells. This activation was not accompanied by enhanced ERK1/2 or MEK1/2 phosphorylation [29]. To further test for the impact of MAPK activity on Tip-mediated SRE-reporter induction, transfected Jurkat T cells were treated with the MEK inhibitors U0126 and PD0325901. PMA, a chemical diacylglycerol analog known to activate MAPK, was included as a positive control for the inhibitory activity of these reagents (Figure 1). Activation of the SRE reporter by Tip was confirmed, but only partially or non-significantly reduced by U0126 or PD0325901 treatment. In contrast, PMA-induced reporter activity, which was 2.8-fold higher compared to Tip-expressing cells, was highly sensitive to MEK inhibition (Figure 1A). These data were concordant with ERK1/2 phosphorylation detected by immunoblot analysis. Basal phosphorylation was rather reduced by Tip, enhanced by PMA and suppressed by the inhibitors (Figure 1C). As Tip-induced SRE activity was not accompanied by MAPK activity, we tested an alternative reporter (Figure 1B). The p3D.A luciferase reporter contains a mutated TCF-binding Ets motif within its SRE and is therefore more sensitive to activation by MRTF:SRF complexes. Relative to the SRE reporter, the p3D.A construct displayed a high basal activity in vector-transfected cells. An enhanced activity of this reporter was observed for the MEK inhibitor U0126, but not for PD0325901, indicating off-target functions and restricting the validity of U0126 data. Tip induced a 3-fold increase of the basal activity, and this enhancement was not significantly affected by the MEK inhibitor PD0325901. In contrast, PMA stimulation of vector-transfected cells enhanced the activity about 7-fold, and this effect was completely abrogated by U0126 and PD0325901. Taken together, the viral oncoprotein Tip induced SRF-responsive luciferase reporters independent of MAPK activity and ERK phosphorylation. Activation of the p3D.A luciferase reporter further points at SRF activation by Tip independent of the MAPK-TCF pathway.

### SRF activation involves actin dynamics and the cofactor MAL

**Figure 2:**
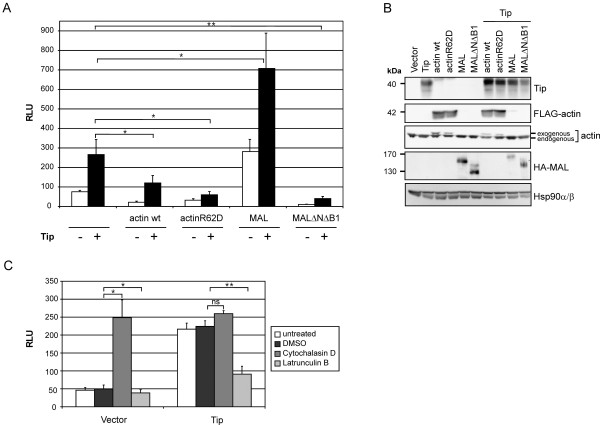
**Effects of actin dynamics and the cofactor MAL on SRF activity**. **(A) **p3D.A-Luc activity of Jurkat T cells transiently transfected with expression plasmids coding for actin wt, actinR62D, MAL and MALΔNΔB1 alone or in combination with a Tip expression construct. **(B) **Representative expression controls carried out by immunoblot analysis with whole cell lysates 48 h post transfection. Detection of Hsp90α/β served as a loading control. **(C) **p3D.A-Luc activity of vector- and Tip-transfected cells treated with Latrunculin B (1 μM) and Cytochalasin D (1 μM) for 24 h. The graphs in (A) and (C) display the mean values and standard deviation of three independent experiments. Statistical significance, p > 0.05 (ns); p < 0.05 (*); p < 0.01 (**)

To corroborate MAPK and, thus, TCF independence of Tip-mediated SRF activation, we next addressed the actin-MRTF pathway. To this end, we transfected Jurkat T cells with expression plasmids for wild-type actin (actin wt), an actin polymerization mutant (actinR62D), wild-type full-length MAL (MAL) and a MAL deletion mutant unable to bind actin and SRF (MALΔNΔB1) alone or in combination with Tip (Figure 2A). Expression of the transfected constructs was controlled by immunoblot analysis (Figure 2B). Overexpression of actin, presumably resulting in excess globular actin, diminished the basal and Tip-induced reporter activity by 3.5- and 2.2-fold, respectively. This effect became more evident when globular actin was enriched by overexpression of actinR62D, which reduced the Tip-induced signal below basal levels. Upon overexpression of MAL, the basal reporter activity was 3.7-fold higher compared to vector alone, and this was further enhanced about 2.5-fold by coexpression of Tip. In contrast, the MAL deletion mutant completely abrogated the signal. To strengthen these observations, we treated transfected cells with Latrunculin B, an inhibitor of actin polymerization and promoter of filamentous actin disassembly. As a positive control we used Cytochalasin D, which binds G-actin irreversibly (Figure 2C). While enrichment of monomeric actin by Latrunculin B inhibited both basal and Tip-induced reporter activity, Cytochalasin D increased the basal activity about 4-fold, but did not further enhance the Tip effect. Thus, actin polymerization and the cofactor MAL indeed play an important role in SRF activation by Tip.

### Dominant-negative Rac1 prevents Tip-mediated MAL:SRF activation

**Figure 3:**
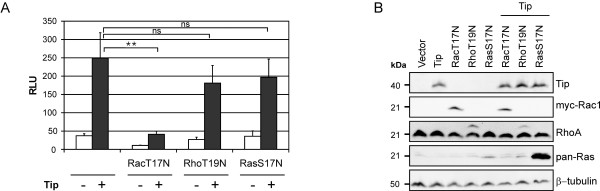
**Influence of dominant-negative GTPases on SRF activation**. Jurkat T cells were transiently transfected with expression plasmids coding for myc-Rac1-T17N (RacT17N), RhoA-T19N (RhoT19N) and H-Ras-S17N (RasS17N) alone or in combination with a Tip expression construct. **(A) **Activity of cotransfected p3D.A-Luc displayed as mean values and standard deviation of three independent experiments. Statistical significance, p > 0.05 (ns); p < 0.01 (**). **(B) **Representative expression control carried out by immunoblot analysis. Detection of β-tubulin served as a loading control

The importance of actin dynamics for Tip-induced SRF activation raised the question whether the small GTPases RhoA, Rac1, Cdc42, inducers of actin polymerization and actin filament stabilization, play a role in this process. Therefore, we used dominant-negative expression constructs for Rac1 (Rac1-T17N) and RhoA (RhoA-T19N) to further elucidate their role in p3D.A reporter induction. Dominant-negative H-Ras (H-Ras-S17N), a regulator of MAPK and TCFs, was used as a control for interference between the small G proteins (Figure 3A). Expression of the transfected constructs was controlled by immunoblot analysis (Figure 3B). Coexpression of RhoA-T19N and H-Ras-S17N did not significantly reduce Tip-mediated reporter activity. However, overexpression of Rac1-T17N impaired both Tip's effect on the reporter and background activity in vector-transfected cells. Effector pull-down assays to detect GTP-loaded Rac1/2/3 and Cdc42 (GST-PAK-CRIB), RhoA (GST-Rhotekin) and H-Ras (GST-Raf-RBD) suggested an activation of Rac and Cdc42, but not RhoA and H-Ras by Tip (data not shown). However, these findings were not constantly reproducible due to high basal levels of activated Rac1/2/3 and Cdc42 in vector-transfected cells. Nevertheless, the luciferase reporter assays demonstrate a major role of the GTPase Rac1, but not of RhoA and H-Ras, in the actin polymerization- and MAL-dependent SRF activation by Tip.

### p3D.A reporter activation by Tip depends on Src-family kinase interaction and activity

**Figure 4:**
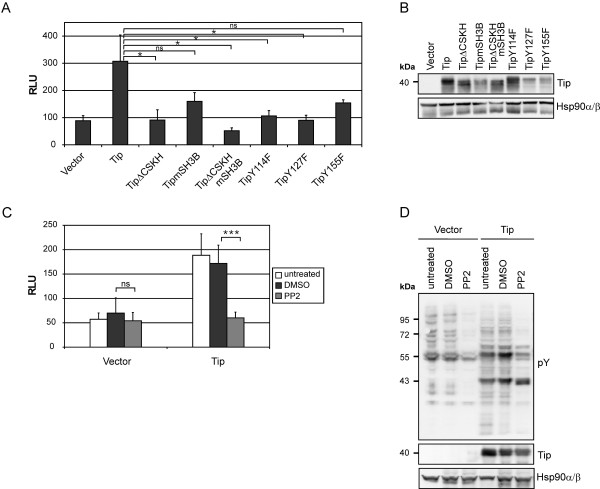
**The role of Src-kinase interactions of Tip for SRF activation**. **(A) **Jurkat T cells were transiently transfected with expression plasmids coding for wild-type Tip or its mutants TipΔCSKH, TipmSH3B, TipΔCSKHmSH3, TipY114F, TipY127F and TipY155F. Empty vector (pEF1) served as negative control. Activation of cotransfected p3D.A-Luc displayed as mean values and standard deviation of three independent experiments. Statistical significance, p > 0.05 (ns); p < 0.05 (*). **(B) **Expression control for Tip and its mutants in total lysates obtained from the cells described in (A). **(C) **Vector and Tip constructs were cotransfected with p3D.A-Luc into Jurkat T cells, and SFK activity was blocked by PP2 treatment (10 μM) for 40 h. The graph summarizes the reporter activities of six independent experiments. Statistical significance, p > 0.05 (ns); p < 0.001 (***). **(D) **Total cellular protein tyrosine phosphorylation (pY) and Tip expression in the cells described in (C). Detection of Hsp90α/β served as a loading control

To test for the properties of Tip required to induce SRF activity, we used mutants of Tip defective in its major effector function, the recruitment and activation of the Src-family kinase (SFK) Lck, or carrying substitutions of the conserved tyrosine residues Y114, Y127 and Y155, which may be targets of Lck [22,27,30] (Figure 4A). Expression of the transfected constructs was controlled by immunoblot analysis (Figure 4B). Deletion of the CSKH motif (TipΔCSKH and TipΔCSKHmSH3B) or individual point mutations of tyrosine residues 114 (TipY114F) and 127 (TipY127F) significantly reduced SRF reporter activity to vector levels. The repression observed upon mutation of the SH3 binding motif (TipmSH3B) or tyrosine residue 155 (TipY155F) was not significant. Furthermore, interpretation of the data for TipY127F and TipY155F is restricted by their expression levels, which were reproducibly reduced relative to the wild-type protein. The abolishment of Tip-mediated reporter activation by the highly specific SFK inhibitor PP2 verified the requirement of Src-kinase activity (Figure 4C). Immunoblot analysis of protein tyrosine phosphorylation monitored a modulating function of Tip and the inhibitory efficacy of PP2 (Figure 4D). Hence, Tip relies on both, Lck interaction and SFK activity, to trigger MAL:SRF reporter activity. Furthermore, tyrosine residues Y114 and Y127, known to be critical for STAT3 activation [28] and IL-2-independent T-cell transformation [22], respectively, likely contribute to Tip-induced SRF activity.

### TCR stimulation induces p3D.A reporter activity

**Figure 5:**
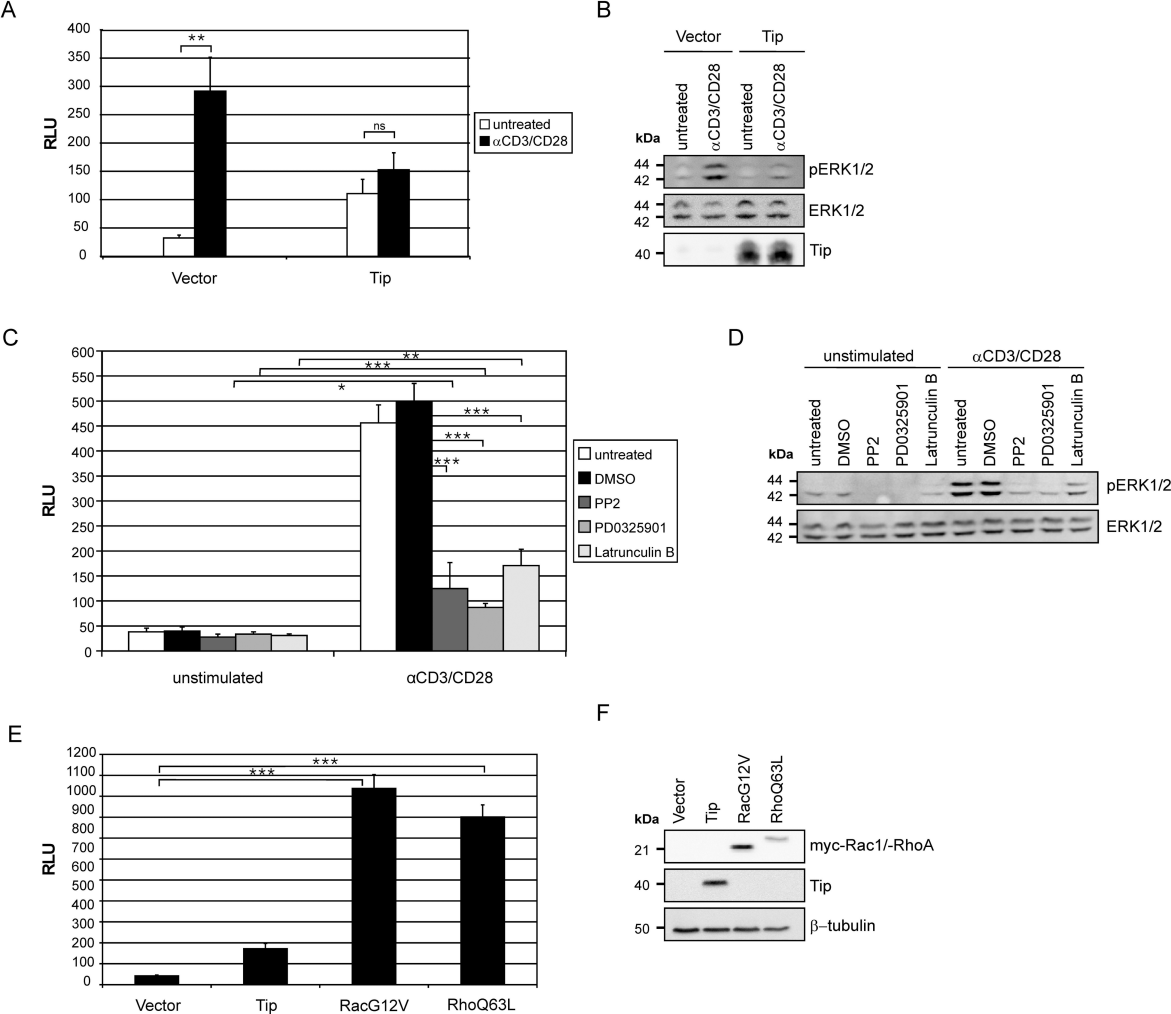
**SRF activation by TCR engagement and by constitutively active Rac1 or RhoA**. **(A) **Jurkat T cells were transiently transfected with p3D.A-Luc and vector or expression plasmids coding for Tip and stimulated for 14 h in 6-well plates coated with antibodies directed against CD3 and CD28 (αCD3/CD28). Reporter activation is summarized as mean values and standard error of five independent experiments. Statistical significance, p > 0.05 (ns); p < 0.01 (**). **(B) **Representative immunoblots for the cells used in (A) displaying ERK1/2 phosphorylation and expression as well as Tip expression. **(C) **Jurkat T cells were transiently transfected with p3D.A-Luc and vector. Cells were treated with PP2 (10 μM, 40 h), PD0325901 (1 μM, 40 h) or Latrunculin B (1 μM, 24 h) and stimulated for 14 h in 6-well plates coated with antibodies directed against CD3 and CD28 (αCD3/CD28). Reporter activation is summarized as mean values and standard deviation of three independent experiments. Statistical significance, p < 0.05 (*); p < 0.01 (**); p < 0.001 (***). **(D) **Representative immunoblots for the cells described in (C) displaying ERK1/2 phosphorylation and expression. **(E) **Vector and expression plasmids coding for Tip, myc-Rac1-G12V (RacG12V) or myc-RhoA-Q63L (RhoQ63L) were cotransfected with p3D.A-Luc. The graph summarizes data of five independent experiments. Statistical significance, p < 0.001 (***). **(F) **Representative immunoblots displaying myc-Rac1-G12V, myc-RhoA-Q63L and Tip expression. Detection of β-tubulin served as a loading control

The viral oncoprotein Tip activated SRF in T cells via the actin-regulated cofactor MAL (Figure 2), while previous reports demonstrated SRF activation via the MEK-ERK pathway in response to TCR stimulation of Jurkat T cells and in mouse T-cell development [13,31]. This discrepancy prompted us to assess whether TCR stimulation alone can trigger the p3D.A luciferase reporter or further enhance the Tip effect (Figure 5A). TCR and coreceptor engagement via CD3/CD28 antibodies resulted in a 10-fold enhanced reporter activity in vector-transfected Jurkat T cells relative to unstimulated cells. In contrast, CD3/CD28 antibody treatment did not significantly augment the Tip-triggered signal. As ERK phosphorylation was absent in Tip-transfected cells (Figure 5B), this lack of cooperation correlated with an impaired CD3/CD28-induced signaling, which is in accordance with suppression of TCR signaling by Tip [32]. In order to specify the TCR-triggered pathway involved, CD3/CD28-stimulated and unstimulated vector-transfected cells were treated with inhibitors of SFK (PP2), MEK (PD0325901), and actin polymerization (Latrunculin B) (Figure 5C). TCR-induced reporter activity was significantly reduced in all treated samples. All three inhibitors were similarly effective, with low but significant residual activities relative to unstimulated cells. Unexpectedly, the residual activities in PD0325901- and Latrunculin B-treated cells did not add up to the activity of solvent-treated cells (DMSO). This finding may be related to the partial reduction of ERK phosphorylation by Latrunculin B (Figure 5D). The impact of actin polymerization on SRF activation in T cells was further addressed by the expression of constitutively active Rac1 (Rac1-G12V) and RhoA (RhoA-Q63L) in the Jurkat system (Figure 5E). Rac1-G12V and RhoA-Q63L (Figure 5E) were equally effective and even more potent than CD3/CD28 stimulation (Figure 5A, C) in inducing 3D.A reporter activity. In conclusion, TCR stimulation relied on both, MAPK signaling and actin polymerization, to activate SRF.

## Discussion

Our study revealed that the oncoprotein Tip of Herpesvirus saimiri (HVS) activates the serum response factor (SRF) in T cells. This activation mainly depends on actin-mediated MRTF coactivation, with minor contributions of MEK-mediated TCF coactivation. Discrimination of coactivator involvement was assessed using two SRF-dependent luciferase reporter constructs, based on the *c-fos *SRE, considered to be specific for TCF coactivation, and on a mutated SRE (3D.A), considered to respond preferentially to MRTF coactivation. However, largely MEK-independent SRE activation by Tip and MEK-sensitive 3D.A activation by PMA revealed a restricted specificity of the reporters in the Jurkat T cells used throughout this study. Hence, we included chemical inhibitors and overexpression of mutant signaling intermediates to assign Tip-induced SRF activation to the actin-dependent MRTF coactivation pathway. Targeting of this pathway by a viral T-cell oncoprotein was unexpected, as SRF function in T cells had previously been linked mainly to the TCF pathway [13].

SRF activation in our system strictly relied on the ability of Tip to engage Lck. This interaction is reported to result in kinase activation [25,33-35], which is also well-known as an initial step in T-cell activation. Indeed, independent of Tip, CD3/CD28 stimulation triggered the 3D.A reporter through Src-family kinase (SFK) activity and both, TCF and MRTF pathways. These findings are in accordance with early results on SRE-dependent transcription in Jurkat T cells [31]. Thus, Lck-dependent MRTF coactivation, which we suggest for Tip, may as well apply to T-cell stimulation. However, while Tip triggers SRF largely independent of MAPK activity, stimulation-induced SRF activation substantially involves MAPK signaling and likely integrates different intracellular signaling routes. The interference of Tip with receptor-mediated SRF activation most likey occurs further upstream. Dependent on its localization in lipid rafts, Tip induces the internalization of TCR complexes [36-38]. Independent of its lipid raft association, Tip blocks TCR-mediated intracellular signaling most likely through sequestration of Lck [32,38]. Consequently, Tip-expressing cells are refractory to receptor ligation by stimulating antibodies.

The dependence of Tip-induced SRF activation on Lck interaction, Src-family kinase (SFK) activity and the potential Lck phosphorylation sites in Tip, Y114 and Y127, draws the attention to the Tip:Lck effectors involved in this pathway. So far, only STATs, especially STAT3, are described as direct targets of Tip-activated Lck [21,24-27]. Tip-induced STAT3 activation depends on residue Y114, which is not required for human T-cell transformation *in vitro *[28]. However, the potential of STAT3 to promote invasion in various cancers [23] may well relate to the massive tissue invasion by HVS-lymphoma cells [26,39], which is not reflected in the cell culture system. Therefore, while effectors of Tip essential for viral T-cell transformation are still not identified, we suggest that Tip Y114 contributes to viral oncogenesis through STAT3-regulated lymphocyte invasion. In this context, STAT3 would be expected as an upstream regulator of RhoGTPases. However, an emerging model positions STAT3 downstream of Rac1 and Cdc42 in the regulation of cell proliferation and migration [40]. Alternatively, transcriptional regulation of genes involved in MRTF:SRF activation by Tip-induced STAT3 appears conceivable. Such an indirect mechanism might also be elicited by STAT5, a recently identified target of Tip [41] likely related to the strict IL-2 dependence of viral transformation in the presence of TipY127F [22]. In any case, a functional link between STAT3 or STAT5 and MRTF:SRF, to the best of our knowledge, has not been reported. Hence, Tip-activated Lck may trigger SRF activation through alternative, yet unknown effectors like the various RhoGTPase guanine nucleotide exchange factors (GEFs) expressed in T cells [42]. Altogether, mechanisms of MRTF:SRF activation proximal to the Tip:Lck complex remain to be established.

The RhoGTPases RhoA, Rac1 and Cdc42 directly regulate actin cytoskeleton organization [7] and therefore share the potential to modulate cellular G-actin pools, which in turn determine MRTF coactivator availability [1]. We expressed constitutively active Rac1 and RhoA and thereby proved the inducibility of MRTF:SRF by both GTPases in T cells independent of Tip. Dominant-negative versions of Rac1, RhoA and Ras were used to test for the involvement of these GTPases in Tip-mediated SRF activation. The missing influence of dominant-negative Ras corroborated the TCF independence of Tip-induced SRF activation. Suppression of the Tip effect by inhibitory Rac1 and not RhoA is in contrast to the initial report on SRF activation by MAL in NIH3T3 fibroblasts [8], but in accordance with MAL signaling in epithelial cells [43]. We assumed that Tip induces SRF via Rac1, but not RhoA. Accordingly, active (GTP-bound) RhoA and H-Ras were not detected in Tip-expressing cells, whereas cellular levels of basally active Rac1 and Cdc42 were enhanced by Tip in some, but not all effector pull-down assays performed. We further used the Rac1/Cdc42-glucosylating *C. difficile *toxins that have been shown to inhibit SRF activation induced by Ca^2+^-dependent dissociation of epithelial integrity [43]. Unexpectedly, the *C. difficile *toxins failed to suppress Tip-induced reporter activity in our Jurkat system (data not shown). This observation is apparently inconsistant with our observation that Rac1-T17N strongly reduces Tip-induced SRF activation. In general, either pronounced Rac1/Cdc42 activation or pronounced Rac1/Cdc42 phosphorylation by Akt1 protects Rac1/Cdc42 from toxin-catalyzed glucosylation and inactivation [44,45]. In particular, protective phosphorylation of Rac1/Cdc42 has to be taken into account, as Jurkat T cells are deficient in expression of PTEN, a major negative regulator of PI3K/Akt signaling [46]. Based on the data available, we would exclude RhoA and Ras and suggest Rac1 and Cdc42 activation in response to Tip expression as the crucial step in SRF induction. The mechanism of the Tip-mediated activation of Rac1/Cdc42, however, remains to be clarified.

Besides the critical role of Rac1 in Tip-induced SRF activation, our results substantiate an essential role of actin and actin-regulated MRTF in SRF activation by Tip in T cells. The syngergism between ectopic MAL and the viral oncoprotein, which is in contrast to the effects obtained with the cellular oncoprotein OTT-MAL [47], points at limiting MAL expression levels and clearly positions Tip upstream in the activation cascade. However, although we used wild-type and mutant MAL expression constructs, our assays are not suited to discriminate the contribution of the individual MRTF-family proteins, MAL/MRTF-A and MRTF-B, which may add another layer of complexity to SRF regulation.

MRTF:SRF functions in T cells are not characterized yet, and T cell-specific target genes of this transcription factor complex are not known. However, transcription of cytoskeletal regulators like MYH9 and MYL9 is elevated in different non-lymphoid cancer cell lines, which depend on MRTFs and SRF for cell spreading, adhesion, and motility [48]. Thus, MRTF:SRF activation by Tip, a viral oncoprotein essential for the development of fulminant T-cell lymphoma characterized by infiltration of multiple organs [16,26], may well contribute to viral oncogenesis and tissue invasion of tumor cells.

## Conclusion

**Figure 6:**
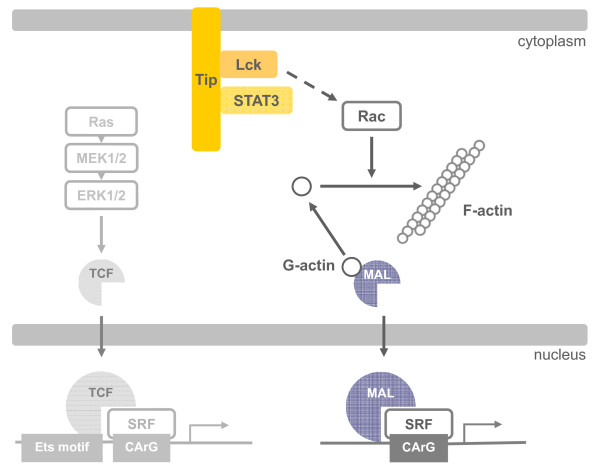
**Model for Tip-induced SRF activation**. The membrane-associated viral oncoprotein Tip engages and activates the Src-family kinase Lck. This interaction is required for STAT3 phosphorylation and for MAL:SRF activation. Signaling flux from Tip:Lck to the RhoGTPases Rac remains to be established (dashed line). Rac promotes a decrease in the G-actin/F-actin ratio and thereby enables nuclear translocation of MAL and coactivation of SRF-dependent gene expression. Tip:Lck is not linked to Ras, MEK1/2, or ERK1/2 and subsequent TCF activation

Our study on cellular signaling by the viral oncoprotein Tip demonstrates SRF coactivation by MRTFs and not TCFs in T cells. MRTF:SRF induction depended on actin polymerization and RhoGTPase activity as well as Tip:Lck interaction and SFK activity (Figure 6). Furthermore, our data hint at MRTF:SRF activation by TCR stimulation independent of Tip. Future studies will have to reveal the detailed mechanisms and target genes of the pathway triggered by Tip as well as its applicability to T cells in general. This approach is anticipated to resolve the functional relevance of MRTF:SRF activity in T-cell regulation and in viral oncogenesis.

## Methods

### Cell culture

Jurkat T cells (E6.1, ATCC TIB-152) were cultured in RPMI 1640 medium supplemented with 10% fetal calf serum (FCS), glutamine (350 μg/ml) and gentamicin (100 μg/ml) at a maximum concentration of 0.5-1 ×10^6 ^cells/ml.

### Transient transfection of Jurkat T cells

Transfection of 5-10 ×10^6 ^cells/ml Jurkat T cells was carried out by electroporation in medium without antibiotics at 250 V, 1,500 μF using a Gene pulser × cell™ Electroporation System (Bio-Rad). For each sample, a total of 50 μg plasmid DNA was used and appropriate empty vector was included to equalize plasmid DNA amounts. Transfected cells, cultured in complete medium without antibiotics, were harvested after 48 h, washed with phosphate-buffered saline (PBS) and processed for luciferase reporter gene assays or immunoblot analysis.

### Expression plasmids

Jurkat T cells were transfected with 20 μg of expression constructs coding for wild type and mutants of the viral oncoprotein Tip derived from HVS-C488: pEF1-Tip, pEF1-TipΔCSKH, pEF1-TipmSH3B, pEF1-TipΔCSKHmSH3B, pEF1-TipY114F, pEF1-TipY127F, pEF1-TipY155F [49]. All Tip constructs are N-terminally myc-tagged. The expression plasmids pEF-FLAG-actin wt; pEF-FLAG-actinR62D, coding for a FLAG-tagged polymerization mutant of actin; pEF-MAL-HA (f.l.), encoding HA-tagged full-length murine MAL; pEF-MALΔNΔB1-HA, coding for a MAL deletion mutant unable to bind to actin and SRF, were described previously [8,50]. Sequences coding for dominant-negative Rac1 (RacT17N) and RhoA (RhoT19N) and constitutively active Rac1 (RacG12V) and RhoA (RhoQ63L) were amplified by PCR with oligonucleotide primers introducing terminal BamHI and EcoRI restriction sites and a N-terminal myc-tag (myc-RacT17N, myc-RacG12V, myc-RhoQ63L) (primers available upon request) were cloned into pEF1 to yield the expression constructs pEF1-myc-RacT17N, pEF1-RhoT19N, pEF1-myc-RacG12V and pEF1-myc-RhoQ63L. Dominant-negative Ras was expressed using the plasmid pcDNA3-RasS17N (kindly provided by A. Wittinghofer, Dortmund, Germany). Integrity of the coding sequences was confirmed by automated DNA sequencing (ABI 3130, Applied Biosystems).

### Immunoblot analysis

Jurkat T cells were lysed in RIPA buffer and processed as previously described to generate whole cell lysates [29]. Protein extracts of 0.5-1 ×10^6 ^Jurkat T cells were loaded on SDS-polyacrylamide gels and transferred to polyvinylidene difluoride membranes (GE Healthcare). After blocking with 5% milk powder in 0.1% Tween20-PBS or NET-gelatine (150 mM NaCl; 5 mM EDTA; 50 mM Tris-HCl pH7.5; 0.05% TritonX-100; 2.5 g/ml gelatine), the membranes were probed with antibodies directed against: phosphotyrosine (4 G10, Millipore), pERK1/2 (pY204 in ERK1), Hsp90α/β (Santa Cruz), ERK1/2, RhoA, Rac1/2/3 (Cell Signaling Technology), Pan-Ras (Calbiochem), Tip [17], Myc-epitope (9E10; ATCC CRL-1729), FLAG-epitope (M2, HRP-coupled; Sigma), HA-epitope (Convance), β-tubulin (GE Healthcare). Binding of primary antibodies was detected using horseradish peroxidase-coupled secondary antibodies directed against mouse or rabbit immunoglobulins (Dako). Primary and secondary antibodies were diluted in blocking buffer. Immunodetection was performed by chemiluminescence and documented with a Kodak Image Station 4000 MM PRO camera.

### Luciferase reporter gene assay

Jurkat T cells were transfected with 20 μg of the individual effector plasmids and 10 μg of the reporter plasmid pSRE-luc containing five SRE of the c-fos promoter (Stratagene) or p3D.A-Luc [51] comprising three SRE with a mutated Ets motif. Cells were harvested 48 h post transfection and divided equally for luciferase activity quantification and immunoblots. For luciferase reporter gene assay, cells were lysed and luminescence intensity was measured as described [52]. Raw data were normalized to the protein content of each sample as determined by a BCA assay (Uptima) and indicated as relative light units (RLU). Data were statistically evaluated with two-tailed t-tests for correlated (Figure 1A) or independent (all other figures) samples using the online-tools provided by the VassarStats Website for Statistical Computation [http://faculty.vassar.edu/lowry/VassarStats.html]. Results were assigned to the categories p > 0.05 (ns, not significant), p < 0.05 (*), p < 0.01 (**), p < 0.001 (***).

### Inhibitor treatment and CD3/CD28 ligation

For inhibitor treatment, transfected Jurkat T cells were seeded in a 12-well plate at a density of approximately 0.5 ×10^6 ^cells/ml. The SFK inhibitor PP2 (Sigma; 10 μM) and the MAPK inhibitors U0126 (Biomol Germany; 25 μM) and PD0325901 (1 μM) were added 8 h post transfection and remained in the cultures until harvesting of the cells. 12-*O*-tetradecanoylphorbol-13-acetate (PMA; Sigma; 20 ng/ml), combined with MAPK inhibitors if applicable, was added for 15 h. To modulate actin polymerization, cells were treated with Latrunculin B (Calbiochem; 1 μM), Cytochalasin D (Applichem; 1 μM) for 24 h. Under these conditions all inhibitors were not toxic to Jurkat T cells as measured by propidiumiodide staining and flow cytometry. T cell-receptor stimulation of transfected Jurkat T cells was carried out for 14 h in a 6-well plate at a density of approximately 1 × 10^6 ^cells/ml previously coated with antibodies against CD3 (OKT3; Janssen-Cilag; 10 μg/ml) and CD28 (a gift from R. Kroczek, Robert Koch-Institut, Berlin; 5 μg/ml).

## Abbreviations

CSKH: Sequence homologous to the C-terminus of Src-family kinase domains; ERK: Extracellular signal-regulated kinase; G-actin: Globular actin; GEF: Guanine nucleotide exchange factor; GTP: Guanosine triphosphate; HVS: Herpesvirus saimiri; MAPK: Mitogen-activated protein kinase; MRTFs: Myocardin-related transcription factors; MYH9: Myosin heavy chain 9: non muscle; MYL9: Myosin light chain 9: regulatory; PMA: 12-*O*-tetradecanoylphorbol-13-acetate; RhoGTPases: Guanosine triphosphatases of the Rho family; SFK: Src-family kinase; SH3: Src homology domain 3 binding sequence; SRE: Serum response element; SRF: Serum response factor; STAT: Signal transducer and activator of transcription; StpC: Saimiri transformation-associated protein of subgroup C; TCF: Ternary complex factor; Tip: Tyrosine interacting protein.

## Competing interests

The authors declare that they have no competing interests.

## Authors' contributions

All authors have read and approved the final manuscript. BB conceived and approved the experiments. KK and JS performed the experiments. KK, SJdJ, JCA, JS, HG, GP and BB analyzed and interpreted the data. BB and KK drafted the manuscript.
